# Tunnel widening in single- versus double-bundle anterior cruciate ligament reconstructed knees

**DOI:** 10.1007/s00167-016-4204-0

**Published:** 2016-06-21

**Authors:** Cathrine Aga, Katharine J. Wilson, Steinar Johansen, Grant Dornan, Robert F. La Prade, Lars Engebretsen

**Affiliations:** 1 Martina Hansens Hospital, Pb 823, 1306 Sandvika, Norway; 2 Orthopaedic Department Oslo University Hospital, Faculty of Medicine, University of Oslo, Oslo, Norway; 3 Oslo Sports Trauma Research Center, Oslo, Norway; 4 The Steadman Clinic, Steadman Philippon Research Institute, 181 W. Meadow Dr. Suite 1000, Vail, CO 81657 USA; 5 Lovisenberg Diakonale Hospital, Pb 4970 Nydalen, 0440 Oslo, Norway

**Keywords:** Tunnel widening, Anterior cruciate ligament, ACL reconstruction, Double bundle, CT, 3D CT, Revision

## Abstract

**Purpose:**

The consequence of tunnel widening after ACL reconstructions is foremost of importance in case of revision surgery. Tunnel expansion leads to bone loss close to the joint, and additional surgery with bone grafting prior to revision surgery might be necessary. The purpose of the study was to measure widening of the tunnels in single-bundle (SB) and double-bundle (DB) ACL reconstructed knees during the first year after surgery, detected by a novel, semi-automated 3D CT imaging modality. Our hypothesis was that there would be a difference between the initial tunnel size and the size measured one year post-operatively due to the tunnel widening process. Further, the purpose was to evaluate whether there were any differences in the amount of tunnel widening between the two surgical techniques.

**Methods:**

Twenty patients who underwent DB ACL reconstruction, and 22 patients who underwent SB ACL reconstruction, performed a CT scan of the bony tunnels, during their first days after surgery and one year post-operatively. The CT scans were transformed into 3D CT reconstructions, and the tunnels were measured with the “best-fit cylinder” method, measurements at the level of tunnel aperture and 10.0 mm from the joint line.

**Results:**

All tunnels in the DB and SB ACL reconstructed knees exhibited widening during the first year after the operation (*p* < 0.001). The SB femoral tunnels showed more widening compared to the DB femoral AM tunnels (1.4 ± 0.9 vs. 0.5 ± 0.6 mm) (*p* < 0.001), and the SB tibial tunnels widened more compared to the DB tibial PL tunnels (1.0 ± 1.0 vs. 0.5 ± 0.6) (*p* < 0.043).

**Conclusion:**

All tunnels widened during the first year after the ACL reconstruction with a larger amount of widening in the SB tunnels compared to the DB femoral AM tunnels and the DB tibial PL tunnels. This is the first study to detect tunnel widening in DB reconstructed knees through a semi-automated 3D CT imaging modality.

**Level of evidence:**

Prospective cohort study, Level III.

## Introduction

Anterior cruciate ligament (ACL) reconstruction tunnel widening tends to occur as an early post-operative finding [23], and there is a wide acceptance that the aetiology behind the enlargement includes both biological and mechanical factors [4, 8]. In the majority of the studies done, no correlation between bone tunnel enlargement and clinical outcomes of the patients has been found [4, 9, 11, 31], although there are also studies where a higher amount of widening has been correlated with increased anteroposterior laxity as measured by the Lachman test and rotational laxity as measured by the pivot shift test [10].

Enlargement of the reamed tunnels in anterior cruciate ligament reconstructed knees can be of significant importance in the case of revision surgery. With more than 120,000 ACL reconstructions performed each year in the USA [19], and with an existing five-year revision rate between 2 and 5 % [2, 16, 22], post-operative ACL reconstruction tunnel widening affects many patients. It consequently may lead to the need for additional surgery with bone grafting.

Various degrees of tunnel widening in ACL reconstructed knees have been reported. Femoral tunnels are known to enlarge between 3 and 45 %; on the tibial side, enlargement from 11 to 45 % has been described [9, 10]. Surgical factors associated with tunnel widening are many: the choice of graft [4, 12, 24, 31], fixation technique and the different fixation devices are all known to have an influence on the phenomenon [6, 15, 20]. It is not clear whether the surgical technique influences tunnel widening. Some articles have studied the widening in knees operated with the double-bundle (DB) ACL reconstruction technique [1, 13, 27], but only a few have compared them to widening in single-bundle (SB) reconstructed patients [1, 10, 11]. Two studies have concluded that there was less widening in the DB operated knees compared to SB, and one study could not find any difference between the two techniques [1, 10, 11].

The DB ACL reconstruction technique is known as a technically demanding procedure with a theoretical higher risk of failure and perioperative complications compared to the SB technique. A complication known with this procedure is the convergence of the two tunnels resulting in tunnel “communication”. This may result in a SB graft tunnel appearance at the femoral or tibial aperture sites [27]. Communication can happen due to the surgical procedure itself, or due to the tunnel widening in the post-operative period [7]. Femoral and tibial reconstruction aimers have been devised to prevent this from happening intra-operatively, but post-operative widening of the tunnels may still occur.

The primary objective of this study was to measure the amount of tunnel enlargement in DB ACL reconstructed knees, detected with a novel semi-automated three-dimensional (3D) computed tomography (CT) measuring method. Our hypothesis was that there would be a difference between the initial tunnel size and the size measured 1 year post-operatively due to the tunnel widening process. Secondary objectives were to measure tunnel widening in SB ACL reconstructed knees and to compare them with the enlargement found in the tunnels of DB ACL reconstructed knees, by using the same 3D CT modality. The last purpose of the study was to detect whether there was any existing tunnel communication at the femoral or tibial side at the time of operation or acquired at 1-year follow-up.

## Materials and methods

During the inclusion period from 2012 until 2014, a prospective cohort of 56 patients out of a total of 120 patients that originally were included in a randomised controlled trial (RCT) comparing the DB and SB surgical techniques (Clinical trials number: NCT01033188), were asked to join the study. Forty-two (20 DB and 22 SB) of the 56 patients were available for the final analysis. The remaining patients were withdrawn either due to logistical issues (*n* = 8) or they did not want to participate due to the additional exposure to radiation (*n* = 1), two patients were lost for follow-up (*n* = 2), and three patients were excluded because of technical errors of the CT scans after image transferring (*n* = 3).

Included patients were between 18 and 40 years with a complete ACL rupture verified by clinical findings, MRI and arthroscopy. Also, they had completed a minimum of 2 months of rehabilitation prior to the operation, and the minimal size of each created hamstring graft bundle was 5.0 mm for the PL bundle and 6.0 mm in diameter for the AM bundle. Exclusion criteria were: previous ACL reconstruction of the affected or contralateral knee, additional injury to other ligaments of the knee which required surgery, meniscal injury leaving less than 50 % of the menisci intact or signs on X-ray of established osteoarthritis (Kellgren–Lawrence grade 3 and 4). If the hamstring tendon grafts were too small and could not obtain a minimal graft diameter of 5.0 mm for the PL bundle graft and 6.0 mm for the AM bundle graft, then the patients were excluded and a randomisation was not performed. The reason for excluding patients with small hamstring grafts, large menisci resections or with established osteoarthritis was because those findings are known to influence long-term outcome of ACL reconstructed knees.

### Surgical technique

The same surgeon (SJ) performed the surgical procedure in all of the included patients. The surgical technique consisted of supine patient positioning on the operating table with the establishment of the regular anterior arthroscopic portals and an accessory anteromedial portal. The ACL tear was confirmed by visualisation and by probing the ACL remnants, and a further debridement of the residual ACL stump and footprints was done. Additional surgery of meniscal or chondral injuries was performed if necessary. The femoral and tibial ACL insertion sites were visualised, and surrounding native soft tissue and bony landmarks were used to define the proximal and distal ACL footprints [33].

### Graft harvesting

An incision was made over the pes anserinus insertion site. The semitendinosus and gracilis tendons were identified and harvested and then doubled or tripled according to their length and thicknesses. The ends of each graft were whipstitched with a non-absorbable suture.

### Single-bundle ACL reconstruction technique (Fig. 1a)

The ACL femoral tunnel was drilled through an accessory anteromedial portal. The centre of the femoral footprint was identified, with the aim to have the tunnel covering both parts of the anteromedial (AM) and posterolateral (PL) bundle attachment sites. With the knee in hyperflexion, reaming of the femoral tunnel was performed with a femoral drill. With a tibial guide, the centre of the tibial tunnel was identified and drilled with a tibia drill sized according to the distal graft size [33]. The graft was passed, and femoral fixation was performed with a suspension device (Endobutton CL, Smith & Nephew, Inc., MA, USA). The tibial fixation was then performed with a non-absorbable interference screw (Biosure PK, Smith & Nephew, Inc., MA, USA).

**Fig. 1:**
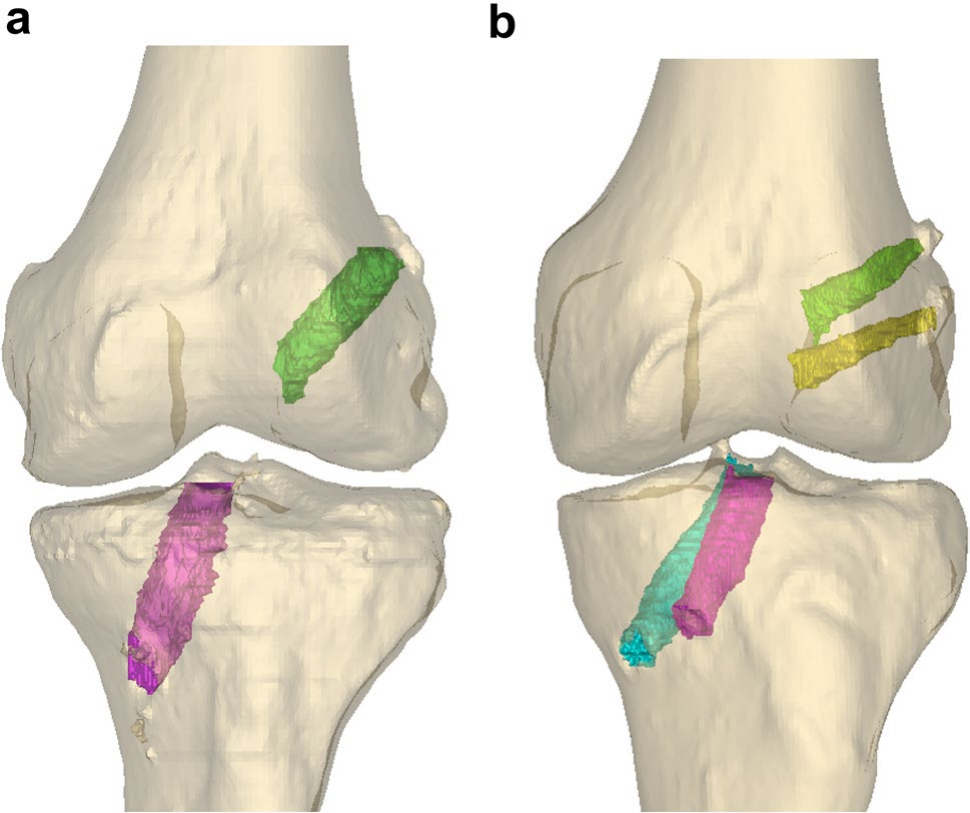
**a** Single-bundle ACL reconstructed knee in a 3D CT model with one tunnel on each side of the joint. **b** Double-bundle ACL reconstructed knee in a 3D CT model with 2 tunnels in the femur AM (*green*) and femur PL (*yellow*) and two tunnels in the tibia AM (*fuchsia*) and tibia PL (*cyan*)

### Double-bundle ACL reconstruction technique (Fig. 1b)

For the DB technique, first the centre of the AM and then the centre of the PL bundle were marked with a Steadman awl through the anteromedial portal. The AM tunnel was drilled first with the knee in a hyperflexed position, and then, the PL tunnel was drilled in sequence, using a DB femoral aimer (Acufex Pinpoint, Smith & Nephew, Inc., MA, USA). At the tibial side, first a tibial aimer was used to drill the AM tunnel, and subsequently, the double-bundle tibial aimer (Acufex Pinpoint, Smith & Nephew, Inc., MA, USA) was used for the PL tunnel. The drill sizes and length of the tunnels were chosen according to the graft sizes. Graft fixation on the femoral side was achieved with a suspension device (Endobutton CL, Smith & Nephew, Inc., MA, USA) for each tunnel. For distal fixation, first the AM bundle was fixed at 70–90° of flexion; then, the PL bundle was fixed with the knee in extension. Both grafts were distally fixated with an interference screw (Biosure PK, Smith & Nephew, Inc., MA, USA).

Notchplasty was not routinely performed and was only realised if any graft impingement existed. Before closure, local anaesthetic was injected in the knee and in the surrounding tissues. The incisions were closed with sutures, and a compression bandage was applied before the tourniquet was loosened.

Immediate free motion and active full weight bearing were allowed from day one. If the menisci were sutured, additional restrictions with 6 weeks of crutches and partial weight bearing were achieved. The physiotherapy included closed chain and isotonic exercises. Running was allowed when muscle strength was adequate, and the patients were advised to wait at least 9 months before return to any pivoting activity. A brace was only applied in three of the patients because of simultaneous medial collateral ligament perforation in order to simplify the meniscal surgery.

## Imaging

The performed software technique for tunnel measurements and the methodology for measuring the tunnels have previously been described in detail [5, 24].

The examination was performed with a 16-row CT scanner (Phillips Brilliance CT 16-slice, Eindhoven, Netherlands) at Oslo University Hospital, Oslo, Norway. The scan consisted of a minimum of 50 image slices, with the following parameters: 1.5 mm slice thickness, 512 × 512 resolution, 120 kVp, 250 mA.

The patients were positioned supine in the scanner with their knee in extension. A continuous scan from the top of the patella down to the tibial metaphysis was performed. Coronal reconstructions were performed to a level parallel to a line joining the posterior femoral condyles; sagittal reconstructions were performed to a level parallel to the outer rim of the femoral condyle. The data were further made anonymous and transferred to the research server of the Steadman Philippon Research Institute, Vail, USA.

### Image processing

All CT images were imported into an image processing software program (Mimics^®^, Materialise, Leuven, Belgium). The bone tunnels were manually segmented first in the axial plane, and then, adjustments were made in the coronal and sagittal planes [24]. A threshold tool was used to create an initial segmentation of each tunnel, and then, manual corrections were made with a stylus pen on a slice-by-slice basis. Each tunnel was reconstructed based on the segmentations and then exported to a 3D modelling software for further analysis (3matic^®^, Materialise, Leuven, Belgium). All segmentations and measurements were performed by the same rater (CA). To measure the inter-rater reliability, 2 additional raters (KW and BC) performed the same segmentation process.

### Measurements of tunnel widening (Figs. 2, 3)

The ends of each tunnel were adjusted to isolate the area of the tunnel where the graft was located and to exclude the suspension device loop. If the tibial screw protruded off to the side of the tibial tunnel on a diagonal, then a line was drawn along the tunnel wall from proximal towards the distal aperture, and the protruding tip of the screw was filtered out. Best-fit cylinder measurements (Fig. 3): A cylinder was fit to the tunnel using a best-fit algorithm within the software, and the diameter and cross-sectional area of the cylinder were recorded [5, 24]. They were compared to the measurements at 1-year follow-up, and the percentage widening was determined.

**Fig. 2:**
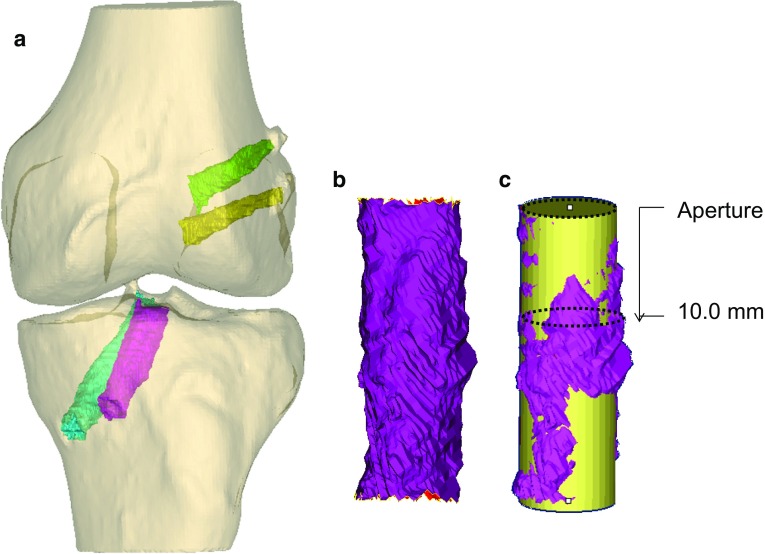
**a** Double-bundle ACL operated knee in a 3D model with the 4 segmented tunnels: femur AM tunnel (*green*), femur PL tunnel (*yellow*), tibia AM tunnel (*fuchsia*) and tibia PL tunnel (*cyan*). **b** The tibia AM tunnel extracted and imported to the 3D-matic software. **c** Measurements of best-fit cylinder (*yellow*); aperture measurements and 10 mm from joint line measurements (*dotted* *lines*)

**Fig. 3:**
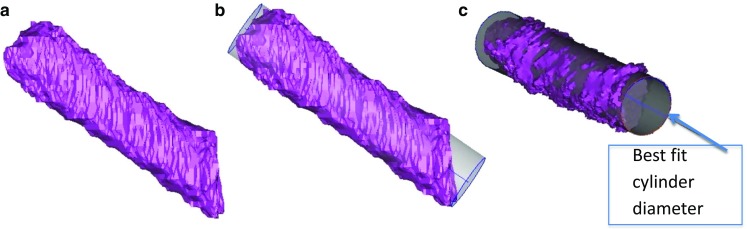
Best-fit cylinder method: The 3D model of the segmented tunnels was exported to the 3D-matic software (**a**). A best-fit cylinder was generated semi-automatically by the software (**b**), and the cylinder diameter was measured (**c**) [5]

Aperture and 10.0 mm level measurements (Fig. 2): The diameter and cross-sectional area at the tunnel aperture and at a distance of 10.0 mm from the aperture at both sides of the joint was measured. The axis of the best-fit cylinder was used to measure the 10.0 mm distance from the aperture. A best-fit circle was then created at the tunnel aperture and at the 10.0 mm distance, and the diameter and cross-sectional area of the circle were recorded at both levels. The diameter and cross-sectional area were compared to the measurements at 1-year follow-up, and the percentage widening was determined.

### Tunnel communication

Communication between tunnels was defined as missing a separating cortical ridge between the two tunnels at the level of the joint line. The distance to the cortical ridge in the affected knees was defined.

Prior to inclusion, all patients signed an informed consent form. All imaging data were made anonymous before the release of scans from Oslo University Hospital. The Research Committee of Imaging at Oslo University Hospital and the Regional Committee for Medical and Health Research Ethics in Norway, approved for the study to be done. Institutional review board approval: ID no 6.2009.234.

To assess measurement reliability, the single measures, absolute agreement definition of the intra-class correlation coefficient (ICC) was calculated with a two-way random effects model. Nonparametric bootstrap confidence intervals were calculated for each ICC measurement. Inter-rater reliability was calculated from 15 randomly chosen subjects (46 tunnels) that were each measured by three observers (CA, KW, BC). The intra-rater reliability was measured based on two rounds of measurement by one investigator (CA), separated by 1 week, for all 15 subjects (46 tunnels). All further tunnel widening analysis was conducted with the first set of measurements of the primary investigator (CA) who was not blinded for the outcome of the patients. The two other observers (KW and BC) were blinded for the patients’ outcome.

### Statistical analysis

An a priori power analysis was performed only for the DB operated knees. Assuming alpha to be 0.05 and a standard deviation of 0.74 mm [28], 20 patients were found to be sufficient to detect a difference of 0.5 mm widening with 80 % statistical power [21].

Paired samples *t*-tests were used to compare the tunnel measurements from 0 to 1-year follow-up in each technique group. Additionally, widening was compared between SB and DB groups using Welch’s two-sample *t*-tests. The chosen level of significance was 0.05. All statistical analyses were performed using the statistical computing software R (R Core Team, Vienna, Austria), including the packages *psy* and *boot*.

## Results

Forty-two cases of primary ACL reconstructed knees were included for analysis. Descriptive data are listed in Table 1. The correlation between the three different observers (inter-rater reliability) was 0.829, 95 % confidence interval (CI) 0.732–0.897. The ICC of repeated measurements of one observer (intra-rater reliability) was 0.963, 95 % CI 0.855–0.988.

**Table 1:** Descriptions of the DB and SB operated patients

	DB (*n* = 20)	SB (*n* = 22)
Age (years)	25.5 [19, 37]	26 [18, 39]
Gender (M/F)	16/4	15/7
Side (L/R)	10/10	12/10
OP till CT1 (days)	0.5 [0, 3]	0.5 [0, 12]
CT1 till CT 2 (days)	366 [333, 460]	367 [337, 767]

Data presented as median [minimum, maximum] or counts

### Difference between initial tunnel size and the size measured one year post-operatively (Table 2)

Every type of SB or DB drilled tunnel exhibited widening from the operation to 1 year post-operatively, except for the DB femoral AM tunnel at the 10.0 mm from joint line level (*p* < 0.05). Results regarding diameter widening of each tunnel are listed in Table 2. Percentage widening of the best-fit cylinders was 7 % (95 % CI = [3, 11]) in the femoral AM tunnel, 14 % (95 % CI = [7, 22]) widening in the femoral PL tunnel, 8 % (95 % CI = [3, 11]) in the tibial AM tunnel and 7 % (95 % CI = [3, 10]) in the tibial PL tunnel. In the SB operated knees, the tunnel/cylinder size widened 17 % (95 % CI = [12, 22]) on the femur side and 10 % (95 % CI = [5, 14]) on the tibia side during the first year after the reconstruction (Table 2).

**Table 2:** Results, tunnel widening Year 0–Year 1

		Year 0 mm ± SD	Year 1 mm ± SD	Widening Year1–Year0 mm (CI)	Widening Year1–Year0 % (CI)	*p* value
*Cylinder measurements*						
SB (*n* = 24)	Femur	8.3 ± 0.6	9.6 ± 1.0	1.4 [1.0, 1.8]	17 [12, 22]	<0.001
	Tibia	10.0 ± 1.0	11.0 ± 1.2	1.0 [0.5, 1.4]	10 [5, 14]	<0.001
DB (*n* = 20)	Femur AM	7.1 ± 0.7	7.6 ± 0.7	0.5 [0.2, 0.8]	7 [3, 11]	<0.001
	Femur PL	5.8 ± 0.4	6.6 ± 1.0	0.8 [0.4, 1.3]	14 [7, 22]	<0.001
	Tibia AM	9.1 ± 0.8	9.8 ± 1.1	0.7 [0.3, 1.0]	8 [3, 11]	<0.001
	Tibia PL	7.0 ± 0.6	7.5 ± 0.8	0.5 [0.2, 0.7]	7 [3, 10]	<0.001
*Aperture measurements*						
SB (*n* = 24)	Femur	8.4 ± 0.6	10.1 ± 1.1	1.7 [1.2, 2.2]	20 [14, 26]	<0.001
	Tibia	9.5 ± 1.0	10.4 ± 1.3	0.9 [0.5, 1.4]	9 [5, 15]	<0.001
DB (*n* = 20)	Femur AM	7.3 ± 0.7	7.9 ± 0.9	0.7 [0.4, 1.0]	10 [5, 14]	<0.001
	Femur PL	5.9 ± 0.4	7.4 ± 1.3	1.5 [0.9, 2.1]	25 [15, 36]	<0.001
	Tibia AM	8.3 ± 0.8	9.2 ± 1.2	0.9 [0.3, 1.5]	11 [4, 18]	0.005
	Tibia PL	6.3 ± 0.6	7.0 ± 1.0	0.7 [0.2, 1.2]	11 [3, 19]	0.004
*10 mm measurements*						
SB (*n* = 24)	Femur	8.3 ± 0.6	9.6 ± 1.4	1.3 [0.7, 2.0]	16 [8, 24]	<0.001
	Tibia	10.1 ± 1.2	11.3 ± 1.4	1.2 [0.7, 1.7]	12 [7, 17]	<0.001
DB (*n* = 20)	Femur AM	7.1 ± 0.6	7.4 ± 0.8	0.3 [-0.1, 0.6]	4 [−1, 8]	ns
	Femur PL	5.8 ± 0.5	6.5 ± 0.8	0.7 [0.3, 1.1]	12 [5, 19]	0.002
	Tibia AM	9.0 ± 0.9	10.0 ± 1.3	1.0 [0.5, 1.4]	11 [6, 16]	<0.001
	Tibia PL	6.7 ± 0.9	7.2 ± 1.1	0.5 [0.1, 1.0]	7 [1, 15]	0.030

### Comparison of tunnel widening for DB versus SB ACL reconstructed knees (Table 3)

There was more widening in the femoral SB tunnel than in the femoral DB AM tunnel when comparing the diameter of the best-fit cylinder. On the tibial side, a significant difference between the tibial SB tunnel and the tibial DB PL tunnel was detected (*p* < 0.05) (Table 3).

**Table 3:** Group comparison of SB and DB

Tunnel	SB widening^a^	Tunnel	DB widening^a^	*p*-value
Best-fit cylinder measurements^a^				
SB femur	1.4 ± 0.9	AM femur	0.5 ± 0.6	<0.001
		PL femur	0.8 ± 1.0	ns
SB tibia	1.0 ± 1.0	AM tibia	0.7 ± 0.8	ns
		PL tibia	0.5 ± 0.6	0.043
Aperture measurements^a^				
SB femur	1.7 ± 1.1	AM femur	0.7 ± 0.6	<0.001
		PL femur	1.5 ± 1.3	ns
SB tibia	0.9 ± 1.0	AM tibia	0.9 ± 1.3	ns
		PL tibia	0.7 ± 1.0	ns
10 mm measurements^a^				
SB femur	1.3 ± 1.4	AM femur	0.3 ± 0.8	0.004
		PL femur	0.7 ± 0.8	ns
SB tibia	1.2 ± 1.1	AM tibia	1.0 ± 1.0	ns
		PL tibia	0.5 ± 1.0	0.037

^a^Data presented as mean diameter (mm) ± SD

Comparing the tunnel widening at aperture in SB to DB operated knees, the only tunnel that showed significant difference in widening 1 year after the operation was the femoral SB tunnel compared to the DB femoral AM tunnel. All other tunnels did not reveal any difference at this level (Table 3). At 10.0 mm from the joint line level, a larger amount of widening was found in the SB femoral tunnel than the DB femoral AM tunnel. On the tibial side, more widening was detected in the SB tunnel compared to the DB PL tunnel (Table 3).

One DB operated knee was identified with communicating tunnels at the first post-operative day. Four additionally DB operated knees were identified with a loss of a separating cortical bridge at 1-year follow-up. Three of the subjects had tunnel communication on the femoral side and two on the tibial side. The distance from the joint line to the cortical bridge was between 2.7 and 15.2 mm.

## Discussion

The most important finding of this study was that a difference in the amount of widening between the tunnels in DB and SB reconstructed knees was found. In two out of three tunnel measurement methods, there was less widening in the DB femoral AM tunnels than in the SB femoral tunnels and in the DB tibial PL tunnels than the SB tibial tunnels.

Another important finding was that this study confirmed the phenomenon of tunnel widening during the first year after the operation, both in DB and in SB reconstructed knees. These findings are consistent with the literature, although the amount of tunnel widening in this study (7–25 %) was less impressive than previously described (Table 4) [13, 27, 29]. The most prominent widening in DB reconstructed knees was found in the DB femoral PL tunnels (12–25 % depending on measurement method).

**Table 4:** Tunnel widening, literature search

Author	Published	Outcome	Patients (*n*)	FU (months)	Imaging	Level of measurement	Widening and clinical outcome**	Results	% Enlargement Femur		% Enlargement Tibia	
L´Insalata et al.	1997	BPTB vs HT	30/30	13/9	X-ray	Widest point	Not studied	HT > BPTB	HT	30.2*	HT	25.5*
									BPTB	NA	BPTB	14.4*
Clatworthy et al.	1999	BPTB vs HT	35/38	12	X-ray	10 mm distal to joint line	No corr	HT > BPTB	HT	40	HT	30.3
									BPTB	NA	BPTB	NA
Jansson et al.	1999	HT	14	24	X-ray MRI	Not specified	Not studied	MRI findings	HT	33	HT	23
Webster et al.	2001	BPTB vs HT	28/33	24	X-ray	Widest part	No corr	HT > BPTB	HT	47.4*	HT	24.1*
									BPTB	15.6*	BPTB	NA
Zysk et al.	2004	BPTB vs HT	7/6	9.5	X-ray	Widest point	Not studied	No diff HT/BPTB	HT	NA	HT	26.7
									BPTB		BPTB	29.9
Fauno et al.	2005	HT	87	12	X-ray	10 mm distal to joint line	Not studied	Extracortical fixation > Joint aperture fixation	HT	NA	HT	NA
Iorio et al.	2007	HT	23	1 day + 10	CT	Aperture, middle point	No corr	Widening femur and tibia	HT	3	HT	11
Järvelä et al.	2008	DB vs SB	32/21	27	MRI	20 mm distal to joint line	Corr to KT1000 and pivot	SB > DB on tibia	DBAM	54	DBAM	39
									DBPL	42	DBPL	43
									SB	45	SB	45
Silva et al.	2010	DB	40	1 day + 3	MRI	Aperture, widest point	Not studied	Widening most midsection, Femoral AM most	DBAM	35*	NA	
									DBPL	30*		
Siebold et al.	2010	DB	24	2 day + 7	MRI	10 mm distal to joint line	Not studied	Widening all tunnels, Femoral PL the most,	DBAM	33.6	DBAM	20.5
									DBPL	46.5	DBPL	38
Kawaguchi et al.	2011	DB vs SB	97/72	24	X-ray	Aperture	No corr	SB > DB	DBAM	7.1*	NA	
									DBPL	0.4*		
									SB	15.5		
Sabat et al.	2011	HT	34	6	CT	Aperture, midway, suspension point	Not studied	Endobutton > Transfix fixation	HT	38.4*	NA	
Lee et al.	2012	DB	40	26.7	MRI	Aperture, mid, exit	Not studied	PL the most Aperture most	DBAM	25.4	DBAM	32.8
									DBPL	30.8	DBPL	44.5
Choi et al.	2013	HT	171	24	X-ray	Proximal, middle, distal	No correlation	EB loop length did not correlate with widening	HT	50.7*	HT	46.4*
Achtnich et al.	2013	DB vs SB	21/24	8	MRI	20 mm from joint line	Not studied	No SB/DB diff	DBAM	41	DBAM	40.6
									DBPL	40	DBPL	43.5
									SB	38.3	SB	40
Robbrecht et al.	2014	Autograft vs Allograft	25/10	12	3D-CT	Best-fit cylinder	Not studied	Allografts > Autografts	Auto	36.7	Auto	35.8
									Allo	53	Allo	47.7
Tajima et al.	2014	DB	51	24	X-ray	Aperture	Not studied	Widening independent of immobilisation period	DBAM	15.2	NA	
									DBPL	14.5		

*NS* not significant

* Largest tunnel diameter, if more than one views (AP or lateral) or measurement point (aperture, midway, exit) was recorded

** Correlation between enlargement of the tunnels and clinical outcome

This is the first study to use a semi-automated 3D CT measuring modality to detect tunnel size changes in DB operated knees. The ICC scores showed an excellent intra-rater and inter-rater reliability [0.963, (95 % CI 0.855–0.988) and 0.829 (95 % CI 0.732–0.897)] making this method reliable and preferable for future studies on tunnel widening. Robbrecht et al. [24] recently published a study with the same measurement method in SB operated knees. They used the best-fit cylinder modality to detect tunnel widening and also reported a high reproducibility with the intra-observer ICC in the femoral tunnels at 0.973 (95 % CI 0.922–0.991) and the inter-observer ICC at 0.992 (95 % CI 0.982–0.996). In the tibial tunnels, the intra-observer ICC was 0.955 (95 % CI 0.875–0.985) and the combined inter-observer ICC was 0.970 (95 % CI 0.987–0.91).

The phenomenon of tunnel widening was found in almost all the measurement modalities (Table 2), although various and often higher degrees of widening in ACL reconstructed knees have previously been described (Table 4). The results of those papers differ substantially (0.4–56 %), as do the modality of imaging, method of measurement, location of measurement along the tunnel and when and what to measure [1, 13, 27]. This has not been consistent when looking at previously described papers (Table 4).

When it comes to the modality of imaging bone tunnels, CT is known to be superior in its reliability. Marchant et al. [17] compared tunnel widening measured on two-dimensional (2D) CT image slices to plain radiography and 2D magnetic resonance imaging and concluded that 2D CT images provided the best inter- and intra-observer reliability and should be used for further evaluation of bone tunnels in patients with tunnel widening [17]. In the current study, 2D CT scans were exported into a 3D model. The benefit of this method was that the tunnels could be extracted after segmentation and the measurements could be calculated semi-automatically using the software. The measurements were also independent of the angulation of the knee at examination. But even though a semi-automated measurement of the tunnels is beneficial, measurement errors due to the manual segmentation technique with this method are still existent and should not be underestimated.

In this study, a post-operative CT scan measuring the tunnel size at time zero and a second CT scan after 1 year were performed. This ensured measuring the real enlargement created only by the post-operative process of the tunnel widening. These results might therefore also be less impressive, though more realistic, compared to studies that did not control for this pre-existent widening at time zero. Iorio et al. [9] looked at widening of single-bundle hamstring tendon grafts. They had one CT scan acquired after the first day of operation and the second one after 10 months. Their results showed 3 % widening of the femoral tunnel and 11 % widening of the tibial tunnel, which is less than other studies but similar to what was found in the present study (Table 2).

Additional measurements were made at the tunnel aperture and 10.0 mm from the joint line on both the femoral and tibial side. Measuring at those two different levels seems of importance in order to detect widening in different parts of the tunnel and to measure where the mechanical and biological forces might have the largest influence on the graft. When comparing the two levels, the results revealed a larger widening at the aperture than 10.0 mm from the joint line for almost all of the tunnels. This is consistent with the previously described literature and has been explained by the windshield wiper effect with graft motion and stress deviation inside the tunnel [25]. The best-fit cylinder measurement method eliminates these irregularities and detects an average widening of the entire tunnel, independent of the different shapes created by the widening.

Multiple studies have been done to compare the DB and the SB reconstruction procedure, and some have found improved knee stability and less graft ruptures with the use of the DB surgical technique [14, 32], though other studies do not have these findings [3, 30]. Our study revealed detectable differences in tunnel widening between the two reconstruction techniques. The femoral AM tunnel and the tibial PL tunnel in DB reconstructed knees had less widening compared to the SB reconstructed knees. Three studies have previously compared widening in DB with SB reconstructed knees. Järvelä et al. [10] looked at 32 DB and 21 SB with MRI 27 months after reconstruction. They reported 39–54 % widening in DB operated knees and a significantly higher degree of widening in SB compared to DB operated knees. The measurements were done 20 mm from the joint line and were compared to the initial drill size. They also found a correlation between clinical laxity and the amount of tunnel widening. Kawaguchi looked at 97 DB and 72 SB operated knees with radiography at 24 months [11]. They only measured at aperture and only at the femoral side and found more widening in SB than DB knees. The widening was only 0.4–7.1 % in DB knees and 15 % in SB operated knees. Achtnich et al. [1] described a widening of the tunnels in both groups, but no significant difference between the widening in the DB and the SB group. The detected widening was between 38 and 44 %. Considering the different results and conclusions in those three studies, both the use of different modalities and methods to detect the widening and the different sample sizes between the studies should be considered. With small study samples like in the studies of Achtnich, Järvelä and the current study, there is a possibility of a statistical type two failure.

Siebold et al. [27] used MRI scans 2 days after the operation and after 7 months and looked at widening in only DB operated knees 10 mm from the joint line on both sides of the joint. They found 20–46 % widening of the tunnels. In their study, the widening was largest around the PL bundle, theorised to be due to the higher non-isometric function of the graft in this position. This is in accordance with the results in this study where the DB femoral PL tunnels had 14 % widening compared to 7 % in the femoral AM tunnels. Both their use of extracortical fixation on both the tibia and the femur and the measurement with MRI instead of CT might influence the results in this study. Considering that two different fixation techniques were used in this study, with an extracortical fixation device at the femoral side and an interference screw fixation at the tibial side, this could influence the results. Tunnel widening is known to increase by extracortical fixation of the graft, compared to fixation close to the joint line [6]. The femoral SB tunnels and the femoral DB PL tunnels had a higher amount of widening compared to their respective tibial side tunnels in the present study (Table 2).

Five of the DB reconstructed knees (20 %) experienced communication at the tunnel aperture 1 year after the operation; 3 on the femoral side; and 2 on the tibial side. Siebold et al. [27] found communication intra-operatively in 4 % of the tunnels, increasing to 23 % after 7 months. It is uncertain to what extent the convergence of the tunnels influences knee function. By creating an anatomic reconstruction of the ACL, a high coverage of the native footprint is desirable [18]. This has been demonstrated to be easier to achieve by the DB technique, and communication of the tunnels at aperture would not influence this. Also the separate directions and tension forces of the two grafts are still ensured, even if the grafts do remain close to each other at the aperture sites.

At revision surgery, the extent of tunnel widening makes an impact. Although the revision rate of DB reconstructed knees is low [3], DB reconstructed knees may be more vulnerable for widening because of the additional bone loss created by the two tunnels on each side of the joint. Thus, tunnel widening might further complicate revision surgery for those knees. The tunnel widening of DB reconstructed knees in this study was between 0.3 and 1.5 mm depending on which tunnel and where the measurements were made. The largest widening was found at the tunnel aperture measurements (1.5 mm). Using the best-fit cylinder method, the largest amount of widening detected was less than 1 mm (0.5–0.8 mm) and thus may not be of importance for the clinical outcome or for the revision procedure.

The main limitation of this study was that it is still uncertain whether these findings affect the patients knee function or if those findings of widening are clinical relevant, because the clinical findings of the patients are yet not available for analysis. Most previous studies did not find any correlation between knee stability and the clinical and subjective outcomes for the patients (Table 4) [4, 9, 11, 31]. Only one study has shown a correlation between widening and knee laxity as measured by KT-1000 on the rotational stability measured by the pivot shift test, with a higher laxity in the patients that were affected of tunnel widening [10]. In a review by Saccomanno et al. [26], five Level 1 or Level 2 studies containing 317 patients were compared. They looked at the clinical and functional outcome of different fixation devices on the femoral side and concluded that the amount of tunnel widening was not found to affect the clinical results.

Other limitations of the present study were the lack of power when looking at the difference between the SB and DB operated knees, because the study sample size only was done to detect a widening in the DB operated knees. Three other studies have looked at the difference between these two techniques [1, 11, 29]. Two of those studies had the same sample size as this study, but to ensure to not overlook any further differences between the two techniques, the groups should have been enlarged. Also, the present study’s results cannot be generalised to other DB reconstructed knees with different graft fixation techniques, other devices or settings. Suspensory devices are known to result in higher amount of tunnel widening than grafts fixated closer to the joint line. As this study has the same fixation technique in both the DB and SB groups, one would suggest that the detected differences would not be influenced, although the amount of widening could be influenced as previously described. Also, a selection and information bias might have occurred, because some of the patients did not want to participate in the study and other patients for different reasons (pain, logistical matters) were not able to obtain the CT scan during the first 2 days after surgery. Finely, no laxity tests were available for analysis at present time. It is therefore not possible to determine whether the reported significant tunnel enlargement could affect knee laxity in our cohort of patients. These results will be available after we have completed the two-year follow-up of all the participants in the trial (Clinical trials number: *NCT01033188*).

The clinical consequence of tunnel widening is first of all of importance in case of revision surgery. Tunnel expansion leads to bone loss close to the joint, and as a consequence to that, additional bone grafting might be necessary before the final revision can be allowed. The extent of tunnel widening is further important in ACL deficient knees reconstructed with the double-bundle technique, because the doubled set of tunnels created with this technique makes them vulnerable for further bone loss.

## Conclusion

In the present study, a higher amount of tunnel widening was found in the SB reconstructed knees compared to two of the four tunnels in the DB reconstructed knees. Further, it was confirmed that widening occurs in all of the tunnels during the first year after surgery. The detected widening was 0.5–1.5 mm in DB operated knees and 0.9–1.7 mm in SB operated knees and was less than previously described. Finally, the novel semi-automated 3D CT method for measuring tunnel widening demonstrated an excellent intra-rater and inter-rater agreement.
